# Allan-Herndon-Dudley Syndrome: A Novel Pathogenic Variant of the SLC16A2 gene

**DOI:** 10.7759/cureus.21771

**Published:** 2022-01-31

**Authors:** Ramin Beheshti, Justen Aprile, Charles Lee

**Affiliations:** 1 Pediatrics, Penn State Health Milton S. Hershey Medical Center, Hershey, USA; 2 Pediatrics, Penn State College of Medicine, Hershey, USA

**Keywords:** genetic syndromes, speech language pathology, thyroid pathology, developmental anomalies, neurotransmitter development

## Abstract

Allan-Herndon-Dudley syndrome (AHDS) is a rare disorder characterized by thyroid irregularities, neurological issues, and developmental delay. In this article, we reported a patient with AHDS who presented with severe developmental delay and failure to thrive in the setting of thyroid irregularities. The patient had missense mutations in the SLC16A2 gene, which codes for monocarboxylate transporter 8 (MCT8). We identified two single-nucleotide variants, including guanine to alanine substitution at position +1 of intron 5 (IVS5+1 G>A) and guanine to alanine substitution at position 1400 of intron 1 (c.1400G>A). This variant has not been previously reported as pathogenic in a patient diagnosed with AHDS, as missense and in-frame single amino-acid deletions have not generally been associated with severe neurodevelopment sequela. We review the clinical and laboratory findings of this rare condition. We will discuss the value of early recognition and diagnosis based on promising clinical trials to treat the neurological and developmental sequela associated with AHDS.

## Introduction

Allan-Herndon-Dudley syndrome (AHDS) is a rare X-linked recessive disease caused by a mutation in the human SLC16A2 gene encoding for monocarboxylate transporter 8 (MCT8) [1]. The MCT8 plays a critical role in transporting thyroid hormones into nerve cells, specifically T3 [2]. Decreased functional MCT8 may lead to intellectual disabilities due to decreased uptake of thyroid hormones by the developing brain [2]. The clinical features include neurological, developmental, and psychomotor disabilities. In addition, the disease may cause thyroid irregularities, including poor weight gain, reduced muscle mass, cold intolerance, constipation, tachycardia (heart rate variability/irregularity), and fussiness [2,3]. The process of myelination and CNS development that peaks in the first two years of life may be severely compromised without the correct transport of thyroid hormones into the CNS [4]. Inability to transport T3 may lead to neurological impairments such as central hypotonia, ataxia, paralysis, aphasia, choreoathetosis, dystonia, non-epileptic paroxysmal movement disorders, hypoplasia, spastic paraplegia, and intellectual incapacities [1]. In addition, an accumulation of T3 peripherally may lead to a thyroid storm [2]. This elevation in free T3 may suppress TSH release [2], which leads to lower levels of TSH and free T4 [2]. The disease has a prevalence of less than 1 in 1,000,000, affecting primarily males [1]. It has been repeatedly reported that the severity of the clinical phenotype is related to the residual transport capacity of the mutated MCT8 protein [1]. Frameshift mutations and large deletions have been identified in severe phenotypes [2]. Missense mutations have not typically been associated with severe phenotypes [2]. Although patients can have varying severity of disease manifestations, it is generally associated with a shortened life expectancy and poor quality of life [1, 2]. There have been only 320 clinical cases reported worldwide since its first official diagnosis in 1944 [5]. We present a case of severe neurodevelopment delay and thyroid irregularities with two single-nucleotide variants in the SLC16A2 gene, including guanine to alanine substitution at position 1 of intron 5 (IVS5+1 G>A) and guanine to alanine substitution at position 1400 of intron 1 (c.1400G>A; p.Val460Ile). These are variants that have not been previously reported as pathogenic in patients diagnosed with AHDS.

## Case presentation

A 14-month-old male firstborn of non-consanguineous healthy parents of Haitian and Jamaican origin was brought in with concerns of failure to thrive and delayed milestones. The child was born via spontaneous vaginal delivery and full-term with no complications. He had an unremarkable immediate postnatal period. Documentations of visits to the primary care physician as early as two months of age for hyperactive symmetric moro and excessive suck reflex, hypotonic upper extremities, hypertonic lower extremities, rhythmic limb movements, constant mouth opening with movement, clenched toes, neck lag, and failure to meet developmental milestones were provided.

One month prior to our encounter, the patient presented to his primary care physician for a routine check-up, where his weight was concerning for failure to thrive (FTT). He weighed 16 lb 12 oz (1 percentile). The patient weighed 16 lbs 15 oz at six months well-child check (WCC). He was referred to a feeding clinic but did not show up to multiple appointments and weight checks. He was drinking 10 ounces of whole milk with an unknown amount of pureed food twice daily. The patient was referred to Children and Youth Services (CYS) at his 11-month WCC visit due to improper feeding, lack of appropriate follow-up, infant weight loss, and lack of parental support. CYS ultimately took custody of the child and placed him with foster parents. At the visit in early February, he was encouraged to take in a minimum of 24 ounces of Pediasure daily in addition to 6-8 oz of pureed foods.

A series of labs were collected and were significant for hypothyroidism. Initial labs included a TSH of 9.8 uIU/mL and free T4 0.5 ng/dL. He was started on Synthroid. Repeat levels two weeks after initial labs were significant for a TSH of 3.26 uIU/mL, free T4 0.7 ng/dL (0.6-1.6 ng/dL), free T3 8.9 pg/mL (2-6 pg/mL), and reverse T3 6 ng/dL (8-25 ng/dL). Though this was in the setting of two weeks of Levothyroxine treatment. Synthroid was discontinued due to concerns of unregulated thyroiditis in the setting of excess triiodothyronine (T3).

**Figure 1:**
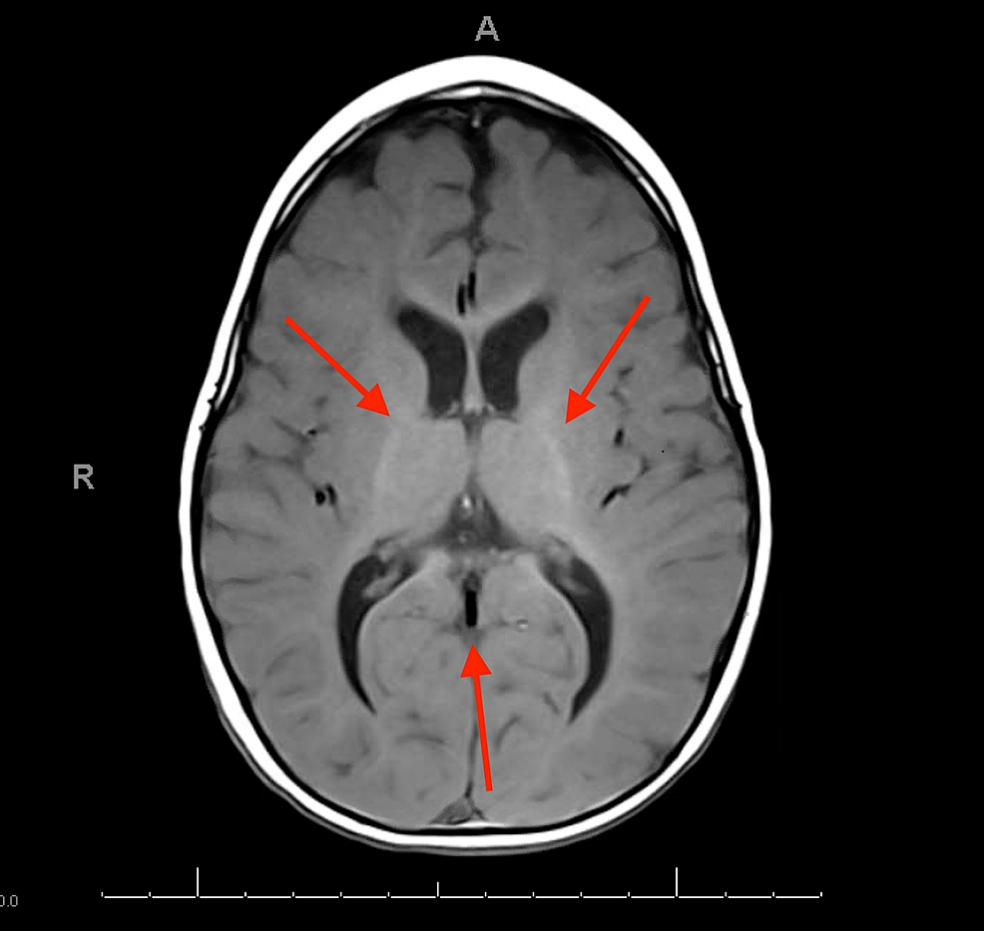
MRI of the brain revealing incomplete myelination with decreased white matter volume and a relatively thin volume of the corpus callosum with ex-vacuo prominence of the ventricles, cisterns, and sulci.

He was transferred to our institution for further evaluation, given continued FTT and repeat lab abnormalities. On physical examination, his vitals were as follows: temperature, 97.8 F (36.5 C); blood pressure, 83/66 mm Hg; heart rate, 124 beats/min; and respiratory rate, 28 breaths/min. Growth parameters were as follows, weight: 9 kg (<1st percentile), length: 77 cm (16th percentile), and head circumference: 47 cm (57th percentile). Exam revealed bi-parietal narrowing. Pupils were equal and reactive with mild ptosis. He had ankle clonus and hypertonicity in all four extremities with central muscular hypoplasia. His patellar and bicep reflex were brisk. The rest of his examination findings were unremarkable. Initial labs revealed an unremarkable complete blood count with differential and comprehensive metabolic panel. Repeat thyroid studies revealed a TSH of 4.45 uIU/mL and free T4 0.79 ng/dL (0.6-1.6 ng/dL), free T3 10.3 pg/mL (2-6 pg/mL), reverse T3 4.6 ng/dL (8-25 ng/dL). MRI of the brain was obtained and revealed incomplete myelination with decreased white matter volume and a relatively thin volume of the corpus callosum with ex-vacuo prominence of the ventricles, cisterns, and sulci (Figure 1).

Blood samples were collected for genomic DNA extraction. In view of developmental delay, FTT, and neurological concerns, clinical genome sequencing and analysis for Cerebral Palsy Xpanded Panel were conducted via Gene Dx. The Cerebral Palsy Xpanded Panel resulted in a hemizygous X-linked mutation with two single-nucleotide variants, including guanine to alanine substitution at position 1 of intron 5 (IVS5+1 G>A), and guanine to alanine substitution at position 1400 of intron 1 (c.1400G>A) of the SLC16A2 gene, which was found to be a likely variant consistent with the diagnosis of AHDS. These specific variants had not been previously reported as pathogenic in a patient diagnosed with AHDS.

## Discussion

Diffuse tissue expression of MCT8 throughout the body may result in a wide spectrum of clinical manifestations [6]. Large deletions in SLC16A2 may result in complete inactivation of MCT8 and a consequently severe phenotype [6]. Most often, large SLC16A2 deletions comprise exon 1, exons 2-4, exons 2-6, exon 3, exons 3-4, and exon 6 [7].

Several missense variants and in-frame single amino acid deletions within the SLC16A2 gene have been associated with milder limitations in MCT8 thyroid hormone transport capacity resulting in a milder clinical phenotype [7]. For example, several missense and in-frame single amino-acid deletions are not generally associated with severe neurodevelopment sequela or thyroid irregularities but have been associated with mild delay in speech development, reading/writing ability, and the ability to walk [7]. However, few missense mutations have been identified with severe pathogenic AHDS [7]. To the best of our knowledge, this case represents the first case of pathogenic AHDS with this specific variant genotype of the SLC16A2 gene. The association of this specific variant and dysthyroidism has not been established. This finding suggests that predicting the phenotypic consequence based on previously identified genotype profiles may not be as clear as previously surmised. Therefore, it is likely that with a better understanding of the pathophysiology, we may be able to delineate the heterogeneity of AHDS and why non-pathogenic variant mutations may lead to more severe clinical phenotypes, such as this case.

An earlier diagnosis may have several benefits from a management standpoint. However, there are currently limited treatment options for this condition, and management is often guided by a multidisciplinary approach [8]. Thyroid irregularities can be regulated by using Levothyroxine and Methimazole to inhibit excessive circulating triiodothyronine. This, however, has not been shown to affect the neurological and developmental disease course [7]. Phase 1 trials of a T3 analog called triiodothyroacetic acid (TRIAC) were found to significantly improve cognition and mobility [8]. This trial demonstrated that TRIAC was safe and effective [8], given the promising results of the clinical trials of the T3 analog, TRIAC. Earlier treatment may lead to improved quality of life and life expectance [8]. Therefore, early awareness and diagnosis may be crucial to this disease process [8].

## Conclusions

Due to its rarity and variable clinical manifestations, AHDS is often underdiagnosed. Given the clinical similarities with more common conditions like cerebral palsy and leukodystrophies, AHDS is often not considered in young children with developmental delays, feeding difficulties, and poor weight gain in conjunction with pyramidal signs, extrapyramidal signs, non-epileptic paroxysmal movement disorders, or ataxia. Additionally, abnormal thyroid studies are typically the only laboratory abnormality that is seen in AHDS. We present a unique SLC16A2 variant that has not been previously reported as pathogenic in a patient diagnosed with AHDS. As missense and in-frame single amino-acid deletions have not generally been associated with severe neurodevelopment sequela, AHDS may be more complex and multifactorial than previously considered, as evidenced by this case.
